# Improving the transition from medical school to internship – evaluation of a preparation for internship course

**DOI:** 10.1186/1472-6920-14-23

**Published:** 2014-02-03

**Authors:** Helen A Scicluna, Michael C Grimm, Philip D Jones, Louis S Pilotto, H Patrick McNeil

**Affiliations:** 1 Faculty of Medicine, University of New South Wales, Sydney, Australia; 2 St George Clinical School, Faculty of Medicine, University of New South Wales, Sydney, Australia; 3 Rural Clinical School, Faculty of Medicine, University of New South Wales, Sydney, Australia; 4 South Western Sydney Clinical School, Faculty of Medicine, University of New South Wales, Sydney, Australia

**Keywords:** Undergraduate medical education, Transition to internship, Outcome based curriculum, Clinical skills

## Abstract

**Background:**

This study evaluates the impact of a new 'Preparation for Internship’ (PRINT) course, which was developed to facilitate the transition of University of New South Wales (UNSW) medical graduates from Medical School to Internship.

**Methods:**

During a period of major curricular reform, the 2007 (old program) and 2009 (new program) cohorts of UNSW final year students completed the Clinical Capability Questionnaire (CCQ) prior to and after undertaking the PRINT course. Clinical supervisors’ ratings and self-ratings of UNSW 2009 medical graduates were obtained from the Hospital-based Prevocational Progress Review Form.

**Results:**

Prior to PRINT, students from both cohorts perceived they had good clinical skills, with lower ratings for capability in procedural skills, operational management, and administrative tasks. After completing PRINT, students from both cohorts perceived significant improvement in their capability in procedural skills, operational management, and administrative tasks. Although PRINT also improved student-perceived capability in confidence, interpersonal skills and collaboration in both cohorts, curriculum reform to a new outcomes-based program was far more influential in improving self-perceptions in these facets of preparedness for hospital practice than PRINT.

**Conclusions:**

The PRINT course was most effective in improving students’ perceptions of their capability in procedural skills, operational management and administrative tasks, indicating that student-to-intern transition courses should be clinically orientated, address relevant skills, use experiential learning, and focus on practical tasks. Other aspects that are important in preparation of medical students for hospital practice cannot be addressed in a PRINT course, but major improvements are achievable by program-wide curriculum reform.

## Background

The preparation of graduates for the transition to internship is the primary responsibility of medical schools [1,2]. Transitions in many occupations are associated with an increased risk of error and it is of paramount importance that medical graduates are well prepared for their transition to internship and that patient safety is assured [3,4]. The University of New South Wales (UNSW) implemented an innovative outcomes-based curriculum in 2004 (referred to as the 'new’ UNSW program). The new program is highly experiential with students involved in clinical experiences in teaching hospitals and community settings from year 1 until graduation [5], and it places explicit emphasis on the learning of generic capabilities needed for professional practice [6]. During the reform process, a 'Preparation for Internship’ (PRINT) course was developed to scaffold graduates’ transition to internship.

As part of the systematic evaluation of the new curriculum, the UNSW Program Evaluation and Improvement Group evaluated the effectiveness of the PRINT course by assessing graduates’ self-perceptions of their clinical capabilities. Evaluating the impact of the PRINT course on graduates’ preparation for internship is complex as the transition is influenced by the graduates’ personal attributes, medical proficiency and the support provided in the hospital environment [7]. Historically, the transition from medical school to internship has been experienced by graduates as stressful and one they do not feel prepared for. Graduates lack confidence in their ability to perform basic skills, they are anxious about managing uncertainty and being responsible for people’s lives [7-13]. Research evidence suggests a discrepancy between the capabilities (knowledge, skills and attitudes) of medical students at graduation and the expectations of their skills by hospital medical, nursing and allied health staff during internship [14-19].

### The Preparation for Internship (PRINT) course

Prior to 2007 UNSW medical graduates who had been enrolled in a relatively traditional content-based curriculum (referred to as the 'old’ UNSW program), were prepared for internship by early exposure to clinical environments, meaningful contact with patients and an accumulation of hands-on clinical experiences integrated throughout the curriculum. A survey undertaken in 2002 of UNSW medical graduates indicated that students felt their training was deficient in several areas relating to internship. Following this, a number of changes were made to the old program. However it was not possible to introduce a dedicated PRINT course immediately due to rigidity in the structure of the existing curriculum. The design of a separate PRINT course to be scheduled after completion of final summative assessments was undertaken as part of the major curricular reform process.

Although PRINT was designed to be the final component of the new outcomes-based curriculum [5,6], it commenced in 2007 as an 8 week course for students undertaking the old UNSW program. PRINT has continued subsequently as a 6 week course for students enrolled in the new program. The 6 week PRINT term comprises attachments to medical and surgical rotations in which students work closely with junior postgraduate doctors and are responsible for the care and management of some patients. It explicitly aims to build on students’ prior clinical learning in clerkships to focus on the capabilities required during internship, by exposing students to the responsibilities of an intern in the assessment and management of patients within a hospital-based health care team. During PRINT, supervisors provide students with feedback on their performance. Students also attend a series of case-method tutorials [20,21], designed to explore some of the common practical, clinical and work-related issues they will encounter as interns. The 2007 and 2009 PRINT courses were structurally the same; the change in length was necessary due to hospital resource allocation.

This paper reports the impact of a new preinternship course at UNSW on medical students’ perceptions of their mastery of clinical capabilities. A comparison of the effect of PRINT on self-perceived capabilities of the two cohorts of students who undertook learning within either content-based or outcomes-based programs provides an evaluation of the utility of an internship transition module, as well as insights into the effects of curriculum reform on preparation for hospital practice. Our use of self-perception data as evaluation metrics are validated by comparison with hospital-based supervisors’ ratings of graduates.

## Methods

### Participants

The participants in this study were the 2007 and 2009 cohorts of UNSW final year medical students who had completed their final summative assessments and were due to commence the PRINT course. The 2007 cohort was from the old medical program, and the 2009 cohort was the first cohort of the new outcomes-based integrated program.

### Procedure

Following research ethics review (UNSW Ethics approval 2007/9/746), students received two emails requesting their participation in an online Clinical Capability Questionnaire (CCQ) prior to and after completing the PRINT course. Medical students also received reminders from clinical school administrative staff encouraging their completion of the questionnaire.

The Hospital-based Prevocational Progress Review Form is completed by hospital-based supervisors and junior doctors at the completion of each 12 week hospital–based rotation [3]. Following research ethics review (UNSW Ethics approval 2009/759) and consent from students, the ratings on the Hospital-based Prevocational Progress Review Form made by hospital-based supervisors and junior medical officers were obtained for 92 graduates from the 2009 cohort who completed their first rotations as junior doctors in March 2010; 73 of these had previously completed the CCQ in 2009.

### Questionnaires

#### The clinical capability questionnaire

The CCQ is a 66 item questionnaire that includes 46 items that evaluate students’ ability to perform clinically-relevant tasks, and 20 items from the Preparation for Hospital Practice Questionnaire (PHPQ), an instrument that has been previously used by medical schools to assess their graduates’ clinical capabilities [22,23]. The 46 clinically-relevant items are divided into 4 subscales that assess capability in clinical skills (18 items), procedural skills (14 items), operational management skills (9 items), and administrative tasks (5 items), with 5 possible responses to the question 'please indicate at which level you believe you can perform the following skills on a patient at the present time’, that ranged from 'I did not try the skill during Medical School’ = 1, to 'I tried the skill and I mastered it’ = 5. A further 20 items assess capabilities in the interpersonal skills, confidence, collaboration and prevention sub-scales of the PHPQ with responses to the question 'please indicate the level at which you believe that medical school prepared you to’ on a 6-point scale ranging from 'very inadequately’ = 1 to 'very adequately’ = 6.

#### The hospital-based prevocational progress review form

The Hospital-based Prevocational Progress Review Form requires supervisors and junior doctors to rate the junior doctor’s performance on 18 questions relating to clinical management, communication and professionalism on a 4 point scale ranging from clearly below the expected level = 1, borderline/requires assistance = 2, at expected level = 3, and clearly above the expected level = 4. A final question rates the junior doctor’s overall performance. A full description of the CCQ and the Hospital-based Prevocational Progress Review Form are published elsewhere [24].

### Analysis

We analysed the data using SPSS version 18. Mean scores for the subscales of both parts of the CCQ were calculated by averaging the raw scores for individual items. T-tests for repeated measures were used to investigate the differences between students’ responses pre-PRINT and post-PRINT for each cohort.

## Results

We received 148 (70%) and 105 (52%) responses from the 2007 and 2009 cohorts respectively prior to completing PRINT and of those 134 (91%) and 73 (70%) responses after PRINT. The lower response rate from the 2009 cohort was possibly explained by student evaluation fatigue, as these students were the first graduating cohort from the new outcomes-based program who had participated in several concurrent evaluation studies. Nevertheless, the demographics of the respondents showed no differences from their respective cohorts. The respondents had a mean age of 24.4 and 23.8 years for 2007 and 2009 respectively, and 57.5% and 62.8% were female.

**Figure 1:**
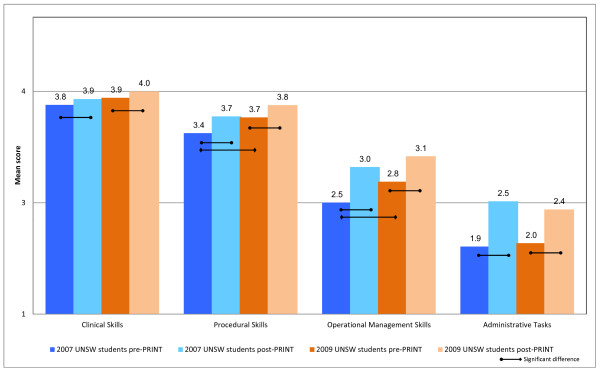
Mean self-perceived capability on 46 clinical tasks of 2009 UNSW outcomes-based curriculum students (orange bars) and 2007 UNSW content-based curriculum (blue bars).

Responses to the 46 clinically-relevant items showed that the mean scores on the clinical skills subscale of the CCQ for the 2007 and 2009 cohorts prior to completing PRINT were 3.8 (SD = 0.4) and 3.9 (SD = 0.4) respectively, indicating that the students perceived that their clinical skills were at a level that they could perform the majority of the clinical skills unsupervised (Figure 1). Students perceived they were progressively less capable completing procedural skills, operational management skills and administrative tasks. The mean pre-PRINT scores on the operational management skills and administrative tasks were 2.5 (SD = 0.7) and 1.9 (SD = 0.8) respectively for the 2007 cohort, and 2.8 (SD = 0.7) and 2.0 (SD = 0.7) respectively for the 2009 cohort, indicating that the students perceived that they could not perform the skill or could only perform the skill when supervised. For these 46 clinically-relevant capabilities, the 2009 cohort of new program students entered PRINT with significantly higher self-perceived capability for procedural (3.7 v 3.4) and operational management skills (2.8 v 2.5) than the 2007 old program students, whereas clinical skill and administrative task capabilities were not different between the two cohorts (Figure 1).

After completing the PRINT course, students in both cohorts perceived their capability with clinical skills, procedural skills, operational management skills and administrative tasks had improved significantly. For example, mean scores on the operational management and administrative tasks had increased from 2.5 to 3.0 and 1.9 to 2.5 for the 2007 cohort and from 2.8 to 3.1 and 2.0 to 2.4 for the 2009 cohort respectively (p < 0.05). The relative improvement from PRINT in each subscale was similar for both cohorts.

**Figure 2:**
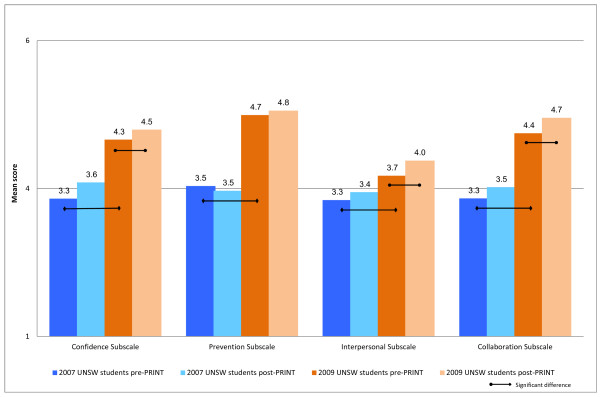
Mean self-reported preparedness for hospital practice of 2009 UNSW outcomes-based curriculum students (orange bars) and 2007 UNSW content-based curriculum students (blue bars) when evaluated pre- and post-PRINT.

Responses to the PHPQ component of the CCQ showed that the 2009 new medical program cohort rated themselves as significantly better prepared for hospital practice than the 2007 old medical program cohort, both before and after PRINT, in the areas of confidence, collaboration, interpersonal skills and prevention (p < 0.05) (Figure 2). There were significant improvements in the post-PRINT ratings for these new program students in 3 out of 4 subscales (confidence, collaboration and interpersonal skills) (p > 0.05) (Figure 2). In contrast, there were no statistically significant improvements in perceptions of preparation for hospital practice by 2007 old medical program students post-PRINT compared with their pre-PRINT perceptions.

**Figure 3:**
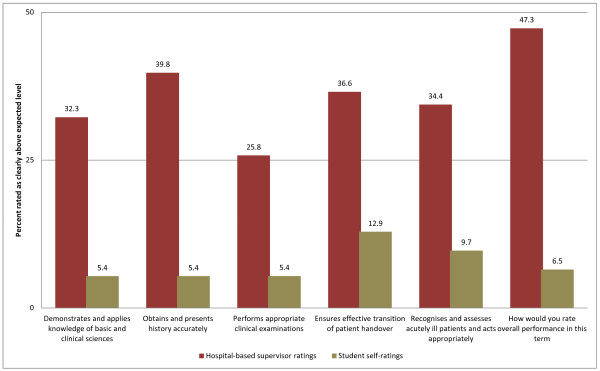
Supervisors’ ratings of UNSW graduates (red bars) compared with graduate self-ratings (tan bars) on questions from the Hospital-based Prevocational Progress Review Form.

To assess the validity of our students’ self-perceptions of capability, the 2009 cohort’s self-ratings of clinical capability were compared to the contemporaneously obtained hospital-based supervisors’ ratings on the Hospital-based Prevocational Progress Review Form. The hospital-based supervisors consistently rated their junior medical officer’s capabilities higher than the junior doctors rated themselves. For example, supervisors rated 47.3% of junior medical officers as clearly above the expected level in their overall performance, whereas only 6.5% of junior medical officers rated themselves at this level of performance (Figure 3).

## Discussion

The difficult nature of the transition from medical school to internship is well documented, as is the lack of intern preparedness as reported by students and their supervisors’ [14,18,19]. To improve the transition from medical school to internship, UNSW developed a PRINT course that was first implemented in 2007 for students who had learned within a traditional content-based curriculum, and continued for subsequent student cohorts who had enrolled in a new outcomes-based program, that places importance on development of generic as well as clinical capabilities [6,24]. Two important conclusions are apparent from our evaluation of this new student-to-intern transition course in these two cohorts.

First, both cohorts had good self-perceived capability in clinical skills prior to commencing PRINT, which reflects that students had received appropriate clinical exposure throughout both 6 year programs, and that they are developing clinical proficiency. The PRINT course made significant but minor improvement in this domain, given the relatively high pre-PRINT capability. The major impact of the PRINT course is a significant improvement in students’ self-perceptions of their capability in procedural skills, operational management skills and intern administration skills, and this was apparent for students from both old (2007) and new (2009) curriculum cohorts (Figure 1). Students perceived their performance improved the most for intern administrative tasks which included skills such as preparing certificates (sick, workers’ compensation, death and cremation) and also obtaining consent for procedures and investigations. The higher hospital-based supervisors’ ratings on the Hospital-based Prevocational Progress Review Form compared with graduate self-ratings confirmed the validity of the students’ self-perception data.

Second, curriculum reform at UNSW has resulted in a major and significant improvement in students’ perceptions of their preparedness for hospital practice, in the areas of confidence, collaboration, preventative medicine and interpersonal skills, and this effect is much greater than any improvement obtained by the PRINT course. Indeed PRINT did not significantly improve self-perceived capability in any of these areas for 2007 old program students, whereas the 2009 new program students did benefit from PRINT in three of the four PHPQ subscales. Of these four capability areas, the interpersonal skills subscale, which includes dealing with difficult, distraught and dying patients or telling a patient they have a terminal illness, had the lowest post-PRINT ratings. Although this is to be expected, as students are likely to have had limited exposure to real life situations involving death and dying, modifications to the Oncology term (year 4) and the Aged Care terms (years 2 and 4) have occurred to further address this difficult area [7,15,25,26].

### Limitations of the study

Prior to undertaking this study the limitations and benefits of evaluating clinical capability using self-perception data were considered. There is mounting evidence that self-perception data is frequently unreliable as participants over-estimate their capacity and are unaware of their errors in judgement [27]. However, there is also evidence that self-perceived capability translates reliably into performance and that self-perception is an important reflective skill in acknowledging strengths and limitations and continuing professional competence [13,19,24,28,29]. Self-report data is also used to guide continuing post-graduate medical education [3,28]. We also considered the literature on the evaluation of undergraduate medical curricula which shows a history of research using self-perception questionnaires, which were posted or emailed to the participants, who were invited to provide self-reports on their performance and that our study would contribute to this chronicle of evaluations [8,13,30-32]. A further consideration was our own resource limitations and the practicalities of obtaining observational clinical reports from clinical supervisors Australia-wide. The use of self-perception data was also consistent with UNSW Program Evaluation and Improvement Group’s evaluation model which values students’ self-perception data as it provides important information to guide change and close the loop in evaluation [33,34]. The comparison of the ratings on Hospital-based Prevocational Progress Review Form made by hospital-based supervisors and junior medical officers indicates that the 2009 UNSW medical graduates generally under-estimated their clinical capacity. It is likely that the same students were able to make accurate and reliable estimates of their clinical performance on the CCQ.

## Conclusions

The results of this study have significant implications for developers of student-to-intern transition courses. Short pre-internship courses such as we have implemented and evaluated are effective in improving clinically-relevant, practical and immediately applicable capabilities that are useful for new graduates to negotiate the transition from student to junior doctor. PRINT was useful for students enrolled in both traditional content-based, as well as newer outcomes-based curricula. However, PRINT courses alone cannot equip graduates with the broader skill sets that are needed to prepare them for hospital practice in the 21^st^ century. Our evaluation indicates that development of these more generic clinical capabilities [6] requires wider curriculum reform. The reader is referred to other sources for a more detailed description of the key aspects of curriculum reform at UNSW [5,6,35]. The results of this study are encouraging as they provide support that the new outcomes-based integrated medical program at UNSW is developing graduates who are clinically competent, skilled communicators and capable team members, and that the PRINT course is effective in equipping students with the practical skills they require for internship.

The model of program evaluation we have implemented at UNSW [33] will encompass continuing evaluation of the PRINT course and our medical program to ensure our graduating students are prepared for both their first position as interns and also for lifelong learning during their medical practice careers.

## Competing interests

The authors report no conflicts of interest. The authors alone are responsible for the content and writing of the paper.

## Authors’ contributions

HAS collected the data, undertook statistical analysis and with HPM analysed the data and drafted the manuscript. HAS, MCG, LSP and PDJ developed the Clinical Capability Questionnaire and edited the manuscript. All authors read and approved the final manuscript.

## Authors’ information

Helen A. Scicluna MEd, PhD is a Senior Lecturer, Faculty of Medicine, University of New South Wales, Sydney, Australia.

Michael C. Grimm MBBS (Hons), FRACP, PhD is a Professor of Medicine and Clinical Associate Dean, St George Clinical School, University of New South Wales, Sydney, Australia.

Philip D. Jones MBBS, FRACP, MHEd, PhD is the Associate Dean (Education) Faculty of Medicine, University of New South Wales, Sydney, Australia.

Louis S. Pilotto MBBS, FRACGP, PhD is Conjoint Professor, Rural Clinical School, Faculty of Medicine, University of New South Wales, Sydney, Australia.

H. Patrick McNeil MBBS, PhD, Grad Dip HEd is a Professor of Rheumatology, South Western Sydney Clinical School, Faculty of Medicine, University of New South Wales, Sydney, Australia.

## Pre-publication history

The pre-publication history for this paper can be accessed here:

http://www.biomedcentral.com/1472-6920/14/23/prepub
